# Advances in microbial sulfoquinovose catabolism

**DOI:** 10.3389/fmicb.2026.1758990

**Published:** 2026-01-27

**Authors:** Yiwei Chen, Dazhi Liu, Ruoxing Chu, Zongyuan Li, Yueya Zhang, Kailiang Ma, Li Jiang, Qiaoyu Yang, Fengxia Lu, Yan Zhang, Yang Tong

**Affiliations:** 1 College of Food Science and Technology, Nanjing Agricultural University, Nanjing, China; 2 New Cornerstone Science Laboratory, School of Pharmaceutical Science and Technology, Tianjin University, Tianjin, China; 3 Meining Pharma, Inc., Tianjin, China; 4 Frontiers Science Center for Synthetic Biology (Ministry of Education), Tianjin University, Tianjin, China; 5 Key Laboratory of Systems Bioengineering (Ministry of Education), School of Chemical Engineering and Technology, Tianjin University, Tianjin, China; 6 State Key Laboratory of Microbial Metabolism, School of Life Sciences and Biotechnology, Shanghai Jiao Tong University, Shanghai, China

**Keywords:** C-C bond cleavage, C-S bond cleavage, sulfoquinovose, sulfonates, sulfoglycolysis, sulfur cycle

## Abstract

Sulfoquinovose (SQ) serves as the polar head group of sulfolipids in photosynthetic organisms. Given the substantial biomass of these organisms, the estimated global annual production of SQ is around 10 billion tons, making it one of the most abundant sulfur-containing organic compounds in nature. The microbial degradation of SQ constitutes a critical component of the global sulfur cycle and is increasingly recognized for its relevance to human health, particularly through its metabolism by the gut bacteria. Microorganisms employ two principal classes of catabolic pathways to degrade SQ: (*i*) C-S bond cleavage pathways, including the sulfo-ASMO (alkanesulfonate monooxygenase-dependent) and sulfo-ASDO (alkanesulfonate dioxygenase-dependent) pathways, which release glucose and sulfite; and (*ii*) C-C bond cleavage pathways, including sulfo-EMP (sulfoglycolytic Embden-Meyerhof-Parnas), sulfo-ED (sulfoglycolytic Entner-Doudoroff), sulfo-TAL (sulfoglycolytic transaldolase), and sulfo-TK (sulfoglycolytic transketolase) pathways, which yield short-chain sulfonates such as sulfolactate (SL) and dihydroxypropanesulfonate (DHPS). These sulfonated intermediates can undergo further degradation, releasing sulfite and short chain carbohydrates. Sulfite-reducing *Bilophila wadsworthia* utilizes them to generate respiratory terminal electron acceptor forming H₂S, which is toxic and a potential cause of inflammation and colon cancer. Here we systematically review the SQ catabolic pathways and the degradation mechanisms of the sulfonated intermediates. In addition, the significant implications of SQ degradation in human gut are discussed briefly.

## 1 Introduction

Sulfonates are abundantly present in environment, food, and even the human body. Their metabolism not only influences the global sulfur biogeochemical cycle, but also is closely linked to human health (Moran and Durham, 2019; Goddard-Borger and Williams, 2017; Roy et al., 2003). Among diverse sulfonates, one of the most abundant organic sulfonate is sulfoquinovose (SQ), which serves as the polar head group of sulfolipid [sulfoquinovosyl diacylglycerol (SQDG)] in photosynthetic organisms and is also present in the N-linked glycans of certain archaea. SQDG is widely found in thylakoid membranes of mosses, algae, ferns, and higher plants and typically accounts for approximately 10% of total lipids (Osawa et al., 2024). It is also found in the membranes of most photosynthetic bacteria (Goddard-Borger and Williams, 2017; Roy et al., 2003; Benson et al., 1959; Haines, 1973; Botte et al., 2011; Anesi et al., 2016; Yongmanitchai and Ward, 1993). SQDG is hydrolyzed by sulfolipase to sulfoquinovosyl glycerol (SQGro), which is then cleaved by sulfoquinovosidase (SQase) (Ma et al., 2025; Li et al., 2024; Kaur et al., 2023). In sulfate-rich marine environments, SQ and other sulfonates produced by phototrophic organisms are subjected to be degraded by heterotrophic bacteria, forming a major component of carbon flux in pelagic ecosystems (Moran and Durham, 2019). Beyond phototrophs, SQ is also present in bacteria like *Bacillus coahuilensis*, which is widely distributed in high-salinity alkaline environments. The ability of this bacterium to supplement membrane phospholipids with sulfolipids is considered an adaptation to sulfur-rich, phosphorus-limited conditions (Alcaraz et al., 2008). Additionally, SQ also forms part of the *N*-linked glycans of membrane-associated protein complexes, surface layer proteins, and filamentous subunits in the acidophilic sulfur-oxidizing archaeon (e.g., *Sulfolobus acidocaldarius*) (Zolghadr et al., 2015). In all these organisms, SQ is synthesized via a NAD^+^-dependent UDP-sulfoquinovose synthase, which catalyzes the reaction between sulfite and UDP-glucose (Zolghadr et al., 2015; Sanda et al., 2001).

SQ has an estimated global annual production of 10 billion tons, which makes it one of the most abundant organic sulfur compounds on Earth. Its degradation is important to terrestrial sulfur cycle and plays a crucial role in the global sulfur biogeochemical cycle (Moran and Durham, 2019; Goddard-Borger and Williams, 2017). SQ is taken up and degraded by diverse bacteria across various habitats. In terrestrial environments, SQ in fallen leaves is metabolized by soil bacteria, accelerating the mineralization of organic sulfur into sulfate, which can then be reabsorbed by plants. Likewise, in lake and marine environments (Chen et al., 2021; Liu L. et al., 2023), SQ is also readily degraded by bacteria. Furthermore, SQ is commonly present in a wide range of plant-based foods in the human diet, including but not limited to vegetables, fruits, grains, legumes, herbs, algae, and edible flowers (Harwood and Nicholls, 1979). The uptake and catabolism of SQ by microorganisms not only provide them with a valuable source of carbon, sulfur, and energy but also promote the recycling of sulfur in the environment. The degradation of SQ by gut microbiota is essential for the utilization of nutrients from food and is closely linked to human health and disease prevention.

Biosynthesis and degradation of SQ are typically segregated among different organisms: phototrophs and certain archaea produce SQ but generally lack complete catabolic pathways, whereas heterotrophic bacteria [notably members of the Roseobacter clade (Chen et al., 2021; Liu L. et al., 2023) and other taxa] specialize in its degradation via diverse sulfoglycolytic or oxidative pathways. Currently, all known SQ degradation pathways are restricted to bacteria and no characterized pathways exist in eukaryotes or archaea, underscoring the central role of bacteria in SQ degradation and driving sulfur cycling in natural ecosystems.

Since the comprehensive reviews by Goddard-Borger and Williams (2017), Snow et al. (2021), and Wei et al. (2022), significant advances have transformed our understanding of microbial SQ metabolism. Recent discoveries have expanded the known catabolic diversity beyond the initially characterized pathways, with the identification of the sulfo-ASDO pathway in marine bacteria, the sulfo-TAL extended pathway in anaerobic gut microbiota (Chen et al., 2024), and the sulfo-TK variant pathway in *Acholeplasma* sp. (Chu et al., 2023). Structural and mechanistic studies have elucidated the catalytic mechanisms of key enzymes, including the radical-based C-S bond cleavage by glycyl radical enzymes (GREs) dihydroxypropanesulfonate sulfolyase (HpsG) (Yan and Liu, 2022) and 6-deoxy-6-sulfo fructose transaldolase (SqvA) (Snow et al., 2023) in sulfo-TAL pathway, and NAD^+^-dependent sulfoquinovose dehydrogenase (SQDH) (Burchill et al., 2025) in sulfo-ED pathway. A novel sulfoquinovosidase (SQase, a member of glycoside hydrolase family GH188) was discovered, it hydrolyzes SQGro to release SQ and is widespread in marine Roseobacteraceae and marine algae (Ma et al., 2025). Furthermore, recent ecological insights have uncovered syntrophic interactions within the gut microbiota, particularly the cross-feeding relationship between SQ-fermenting bacteria *Agathobacter rectalis* and sulfite-reducing pathobiont *Bilophila wadsworthia*, which converts sulfonated intermediates dihydroxypropanesulfonate (DHPS) into hydrogen sulfide through terminal respiration (Hanson et al., 2021). This metabolic axis has profound implications for understanding diet-microbiota-host interactions and their roles in intestinal health and disease.

**Figure 1:**
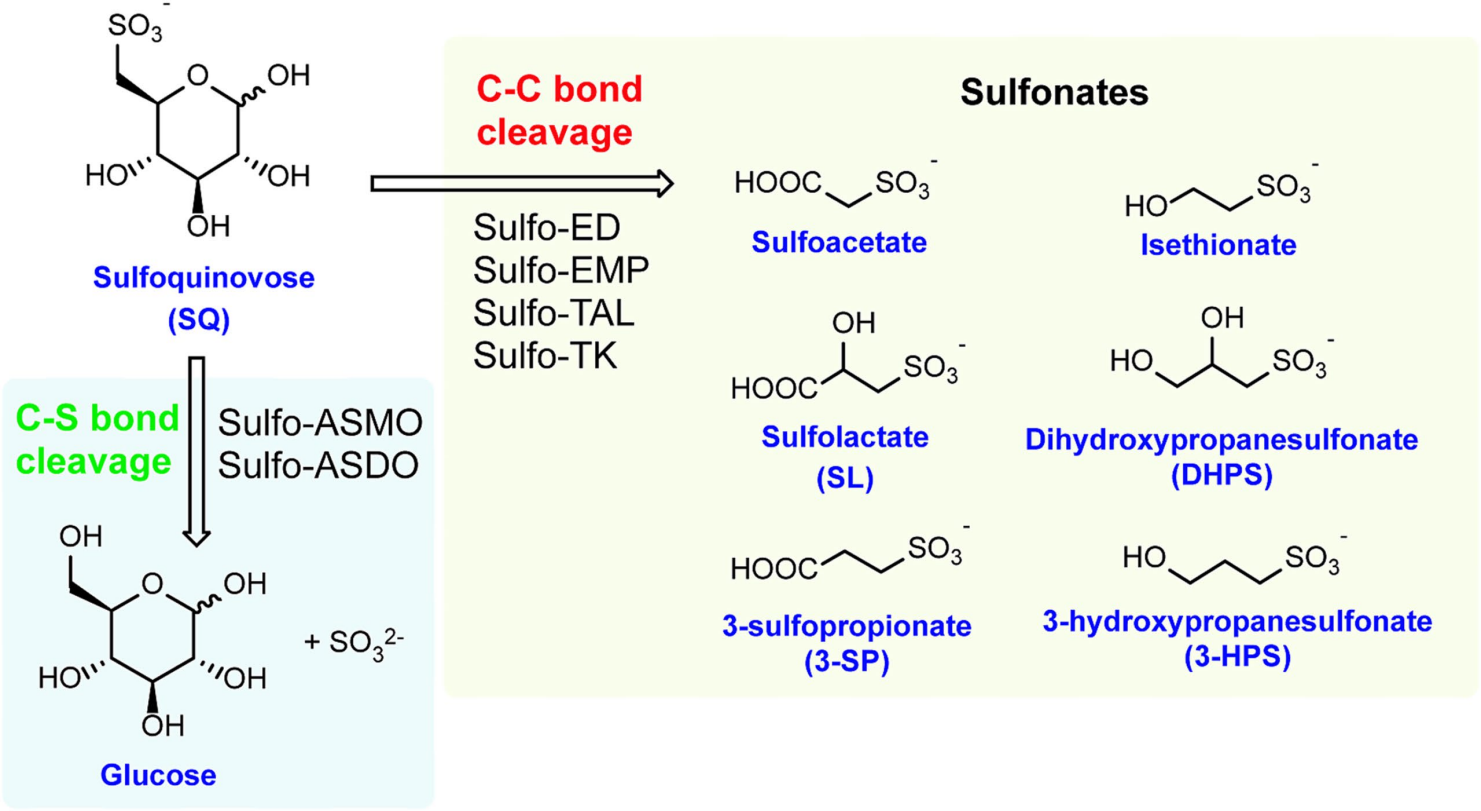
Summary of the SQ catabolic pathways. SL, Sulfolactate; DHPS, dihydroxypropanesulfonate; 3-SP, 3-sulfopropionate; 3-HPS, 3-hydroxypropanesulfonate; sulfo-ASMO, sulfoglycolytic sulfoquinovose monooxygenase pathway; sulfo-ASDO, sulfoglycolytic sulfoquinovose dioxygenase pathway; sulfo-EMP, sulfoglycolytic Embden–Meyerhof–Parnas pathway; sulfo-ED, sulfoglycolytic Entner–Doudoroff pathway; Sulfo-TAL, sulfoglycolytic sulfofructose transaldolase pathway; Sulfo-TK, sulfoglycolytic sulfofructose transketolase pathway.

To date, we know that the degradation of SQ primarily occurs via two types of pathways: C-S bond cleavage pathways that utilize oxygenase generating dehydroglucose, followed by reduction to glucose and C-C bond breaking pathways that produce short-chain sulfonated carbohydrates, which require additional desulfurization steps. (Figure 1) (Snow et al., 2021; Chen et al., 2024). This review provides a comparative analysis of these pathways and briefly summarize recent advances in understanding SQ degradation by gut microbiota. Elucidating these catabolic mechanisms will also facilitate the efficient utilization of SQ in biomass fermentation processes.

## 2 C-S bond cleavage pathways of SQ

The only difference between glucose and SQ lies in the substitution of C-6 hydroxyl group with a sulfonic acid moiety. Oxidative cleavage of the C-S bond, followed by reduction, yields glucose. Glucose can subsequently undergo further catabolism via pathways such as glycolysis or the pentose phosphate pathway. Researches have elucidated two metabolic pathways, through which SQ is converted to glucose via C–S bond cleavage.

### 2.1 The sulfo-ASMO pathway

The sulfo-ASMO pathway identified in *Agrobacterium tumefaciens* (Sharma et al., 2022) and *Novosphingobium aromaticivorans* (Liu et al., 2021) enables complete utilization of carbon atoms from SQ through cleavage of its C-S bond, concomitantly releasing sulfite. It was named according to the alkane sulfonate monooxygenase that catalyzes the oxidative C-S bond cleavage reaction.

**Figure 2:**
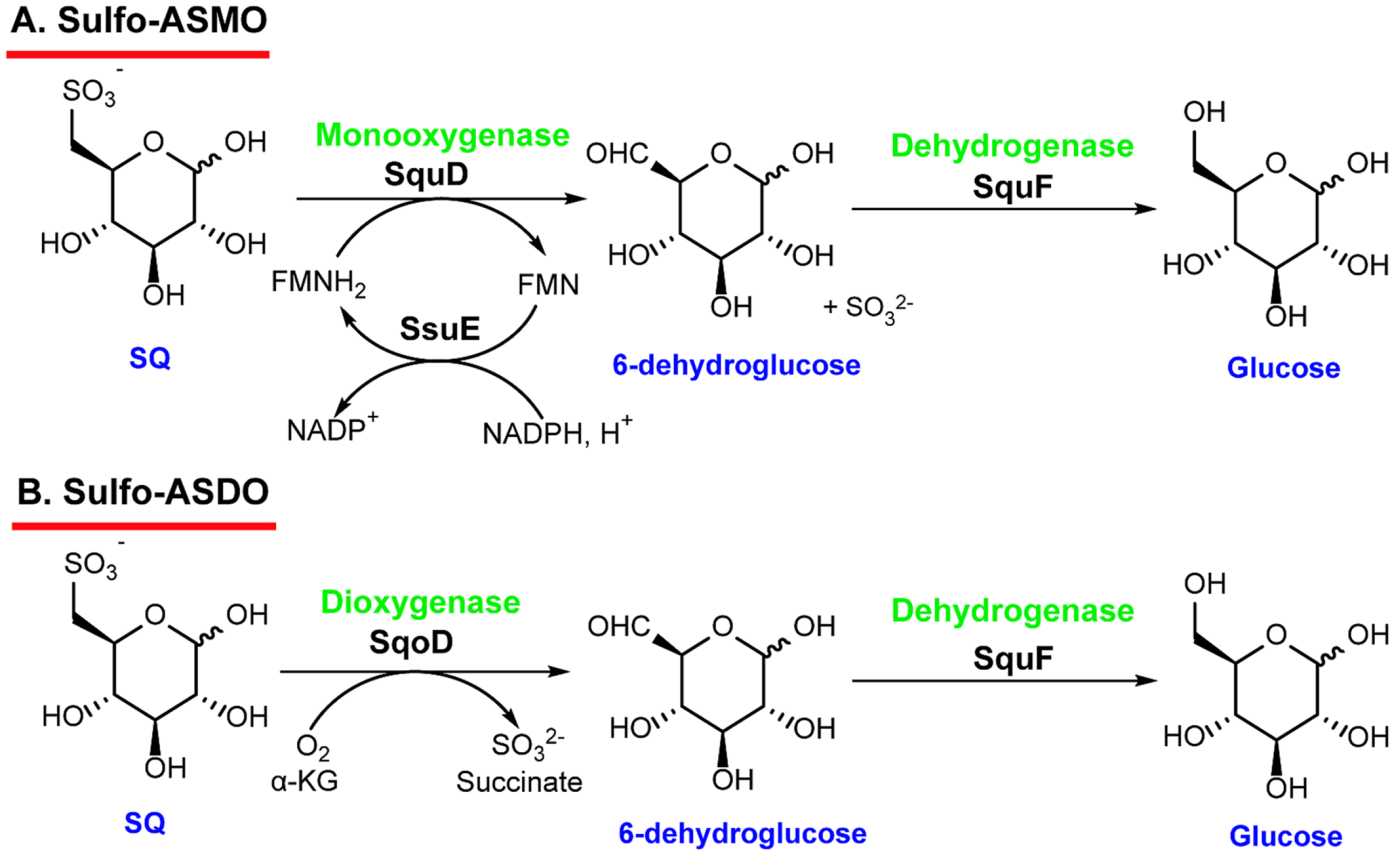
C-S bond cleavage pathways that generate glucose and sulfite. **(A)** The sulfo-ASMO pathway, initiated by alkanesulfonate monooxygenase, was identified in *Agrobacterium tumefaciens* and *Novosphingobium aromaticivorans*. **(B)** The sulfo-ASDO pathway, initiated by alkanesulfonate dioxygenase, was identified in *Marinobacterium aestuarii*.

In the sulfo-ASMO pathway, the degradation of SQ is initiated by the flavin-dependent alkane sulfonate monooxygenase SquD, which converts SQ to 6-dehydro-D-glucose and sulfite by an oxidative C-S bond cleavage. Usually, SquD and SsuE form a two-component monooxygenase system, in which the NAD(P)H-dependent flavin reductase SsuE is responsible to regenerate reduced flavin (FMNH₂) (Liu et al., 2021). Following the C-S cleavage by SquD/SsuE, the NAD(P)H-dependent oxidoreductase SquF reduces 6-dehydro-D-glucose to form glucose, which can enter primary metabolism (Figure 2A). As the sulfo-ASMO pathway requires oxygen for C-S bond cleavage, it is exclusively present in aerobic bacteria, mainly from the *α*- and *ß*-Proteobacteria (Sharma et al., 2022; Liu et al., 2021).

### 2.2 The sulfo-ASDO pathway

An alternative pathway for C-S bond cleavage of SQ, the sulfo-ASDO pathway has been identified in *Marinobacterium aestuarii* and designated by the alkanesulfonate dioxygenase involving in the C-S bond cleavage step. Like the sulfo-ASMO pathway, SQ undergoes oxidative cleavage of the C-S bond catalyzed by the iron and *α*-ketoglutarate-dependent SQ dioxygenase SqoD (Ye et al., 2023), resulting in the formation of sulfite and 6-dehydro-D-glucose, which is then reduced to glucose by the NAD(P)H-dependent oxidoreductase SquF (Figure 2B), while sulfite is excreted as an end-product (Ye et al., 2023).

## 3 C-C bond cleavage pathways of SQ

It has been revealed that, in addition to catabolic pathways involving C-S bond cleavage, SQ can also be degraded via C-C bond cleavage to yield short-chain carbohydrates containing a sulfonate group. This process is accompanied by the generation of different short-chain carbohydrates. To date, four distinct C-C bond cleavage pathways for SQ degradation have been identified in bacteria.

### 3.1 The sulfo-EMP pathway

**Figure 3:**
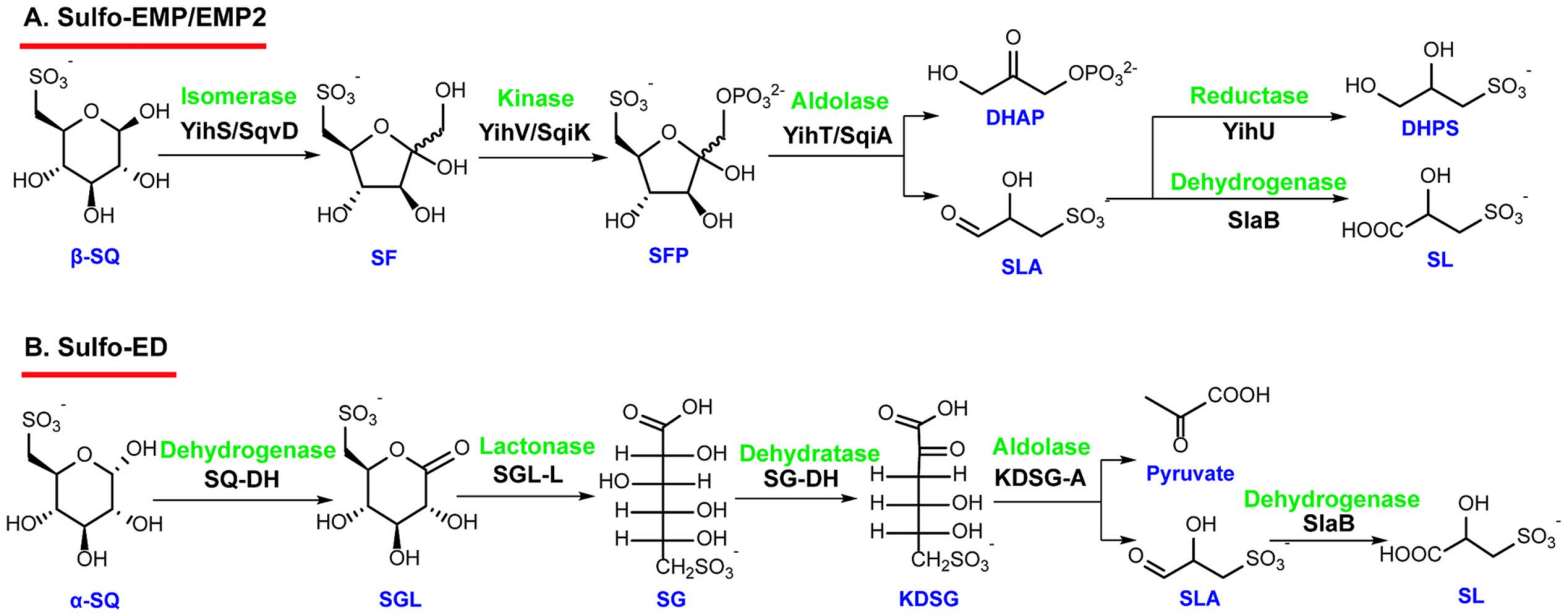
The Sulfo-EMP/EMP2 and Sulfo-ED pathways. **(A)** The sulfo-EMP and sulfo-EMP2 pathways have the same catabolic route but using two different sets of enzymes. Sulfo-EMP was identified in *Escherichia coli*, *Arthrobacter* spp., and related Actinobacteria. Sulfo-EMP2 was identified in *Bacillus urumqiensis* and *Megamonas rupellensis*. **(B)** The sulfo-ED pathway is catalyzed by enzymes that share significant homologies with those of the ED glycolytic pathway. It was identified in *Pseudomonas putida* SQ1 and *Rhizobium leguminosarum* SRDI565. SF, sulfofructose; SFP, sulfofructose-1-phosphate; DHAP, dihydroxyacetone phosphate; SLA, sulfolactaldehyde; DHPS, dihydroxypropane sulfonate; SL, sulfolactate; SGL, 6-deoxy-6-sulfogluconolactone; SG, sulfogluconate; KDSG, 2-keto-3-deoxysulfogluconate.

The sulfoglycolytic Embden-Meyerhof-Parnas (sulfo-EMP) pathway was the first discovered sulfosugar degradation pathway. Researches by Denger et al. (2014) on *Escherichia coli* K-12 MG1655 firstly demonstrated that microorganisms degrade SQ via a mechanism analogous to the EMP pathway (Erlandsen et al., 2000). The enzyme reactions involved in this pathway exhibit considerable analogy with those of the classical glycolytic EMP pathway (Ronimus and Morgan, 2003; Ayna and Moody, 2020). Their studies revealed that SQDG is hydrolyzed by sulfolipase and the sulfoquinovosidase YihQ to produce the *α*-sulfoquinovose (*α*-SQ) isomer (Speciale et al., 2016), which is converted into *β*-sulfoquinovose (*β*-SQ) by the sulfoquinovose mutarotase YihR (Abayakoon et al., 2018). Subsequently, *β*-SQ is isomerized into 6-deoxy-6-sulfofructose (SF) by the aldose-ketose isomerase YihS (Sharma et al., 2021). The resulting 6-deoxy-6-sulfofructose is then phosphorylated by the sulfofructose kinase YihV to form sulfofructose-1-phosphate (SFP), which is cleaved by SFP aldolase YihT to generate dihydroxyacetone phosphate (DHAP) and sulfolactaldehyde (SLA) (Sharma et al., 2021). The resulted DHAP enters central metabolic pathways for further degradation, while SLA is reduced to dihydroxypropanesulfonate (DHPS) by the NADH-dependent reductase YihU (Figure 3A) (Sharma et al., 2020). This sulfonate is subsequently exported from the cell via the major facilitator superfamily (MFS) transporter YihP (Denger et al., 2014; Burrichter et al., 2018; Denger et al., 2012).

Recently, Kaur et al. (2022) showed that the sulfo-EMP pathway also exists in gram-positive *Arthrobacter* spp. and related Actinobacteria. Interestingly, these bacteria possess genes encoding the homologue enzymes of YihS, YihV, and YihT, but lack genes for YihQ and YihR. Despite this, they retain the ability to utilize sulfoquinovoside and methyl α-sulfoquinovoside, suggesting the involvement of unknown or non-specific sulfoquinovosidases in the catalysis. Furthermore, studies have revealed that the sulfolactaldehyde reductase in these bacteria is replaced by a sulfolactaldehyde dehydrogenase, SlaB. Instead of being reduced to DHPS, SLA is oxidized by SlaB via an NAD^+^-dependent reaction to generate sulfolactate (SL) (Figure 3A), which is subsequently exported out of the cell (Kaur et al., 2022). It was proposed that, in the sulfo-EMP pathway, facultative anaerobic fermentative bacteria such as *E. coli* preferentially consume NADH to form DHPS, whereas *Arthrobacter* species and related aerobic bacteria favor a pathway that generates NADH, leading to the production of SL (Wei et al., 2022).

In addition, through bioinformatic analysis of the gene clusters containing sulfoquinovosidase gene *yihQ*, Liu et al. identified a variant of the sulfo-EMP pathway—termed the sulfo-EMP2 pathway—in the aerobic bacterium *Bacillus urumqiensis* and the anaerobic bacterium *Megamonas rupellensis*. Both bacteria belong to the phylum Firmicutes. Compared to the canonical sulfo-EMP pathway, the sulfo-EMP2 pathway degrades SQ through the same metabolic intermediates, including 6-deoxy-6-sulfofructose, sulfofructose-1-phosphate, and SLA, and ultimately generates the same sulfonate byproducts—either DHPS or SL via the reduction or oxidation of SLA. However, the enzymes involved in the sulfo-EMP2 pathway, including SqvD, SqiK, and SqiA, are non-orthologues of the sulfo-EMP pathway. They share low sequence homologies with their isozymes, YihS, YihV, and YihT. The sulfo-EMP2 pathway is predominantly found in gram-positive bacteria of Firmicutes phylum, which implies a convergent evolution phenomenon of this metabolic route among distinct microbial lineages (Liu et al., 2021).

Sulfo-EMP1 and sulfo-EMP2 pathways employ class I (YihT) and class II (SqiA) SFP aldolases, respectively. These two classes of aldolases show mutually exclusive distributions in gram-positive bacteria: class I is found in Actinobacteria, while class II is present in Firmicutes. In gram-negative Proteobacteria, both classes co-occur. Correspondingly, the sulfo-EMP1 pathway is exclusively present in Proteobacteria and Actinobacteria, whereas the sulfo-EMP2 pathway is found only in Firmicutes. Hybrid sulfo-EMP1/2 protein combinations are distributed across Proteobacteria and Actinobacteria (Sharma et al., 2023). This demonstrate that how different bacterial phyla utilize distinct chemical approaches to metabolize SQ, emphasizing its significance as a carbon source.

The fate of the central intermediate SLA is dictated by the competing kinetics of redox enzymes, which directs carbon flux toward either excretion (as DHPS/SL) or energy conservation. The divergence between the sulfo-EMP/reductive (DHPS-forming) and sulfo-EMP/oxidative (SL-forming) pathways is largely controlled by the properties of the SLA-utilizing enzymes. The NADH-dependent sulfolactaldehyde reductase (YihU), common in fermentative anaerobes like *E. coli*, exhibits high catalytic proficiency (*k*_cat_ = 332 ± 9 s^−1^, *K*_M_ = 0.3 ± 0.038 mM, *k*_cat_/*K*_M_ = 1,090 ± 120 mM^−1^ s^−1^) for SLA reduction (Sharma et al., 2020), favoring the formation of DHPS to regenerate NAD^+^. Conversely, the NAD(P)^+^-dependent sulfolactaldehyde dehydrogenase (SlaB/GabD), found in aerobes like *Rhizobium leguminosarum*, catalyzes the oxidation of SLA to sulfolactate (SL). Kinetic analysis of SlaB reveals a *K*_M_ for SLA in the range of 0.09–0.17 mM, a *k*_cat_ of 17.8 ± 7.0 s^−1^, and a *k*_cat_/*K*_M_ of 137 ± 72 mM^−1^ s^−1^, driving the flux toward SL production for excretion or further catabolism (Li et al., 2023).

### 3.2 The sulfo-ED pathway

The second sulfosugar C-C bond cleavage pathway is known as the sulfoglycolytic Entner–Doudoroff (sulfo-ED) pathway (Felux et al., 2015). It was initially identified in *Pseudomonas putida* SQ1 (Felux et al., 2015), and later described in studies of the plant symbiont *Rhizobium leguminosarum* SRDI565 (Li et al., 2020). The enzymes involved in this pathway share significant homologies with those of the classical ED glycolytic pathway (Conway, 1992).

In this pathway, *α*-SQ is first oxidized to 6-deoxy-6-sulfogluconolactone (SGL) by a NAD^+^-dependent sulfoquinovose dehydrogenase (SQDH). SGL is then hydrolyzed and ring-opened by an SGL lactonase (SGL-L), yielding 6-deoxy-6-sulfogluconate (SG). Subsequently, SG undergoes dehydration catalyzed by 6-deoxy-6-sulfogluconate dehydratase (SG-DH) to form 2-keto-3,6-dideoxy-6-sulfogluconate (KDSG), which is then cleaved by a dedicated KDSG aldolase (KDSG-A) to generate pyruvate and SLA (Figure 3B). The resulting pyruvate can be further oxidized to acetyl-CoA and enter the tricarboxylic acid (TCA) cycle for additional energy metabolism, while SLA is oxidized to SL by SlaB, and then exported out of the cells. Gene cluster analyses suggest that the sulfo-ED pathway is primarily present in soil-dwelling Gram-negative Proteobacteria, particularly within the *α*-, *ß*-, and *ɤ*-proteobacteria classes (Felux et al., 2015).

The upstream enzyme SQDH acts as a metabolic gatekeeper. Structural and kinetic studies of *Pseudomonas putida* SQDH demonstrate a stringent preference for NAD^+^ over NADP^+^, with a catalytic efficiency (*k*_cat_/*K*_M_) approximately 10^6^-fold higher for NAD^+^ (Burchill et al., 2025). This specificity ensures that the oxidative steps of sulfoglycolysis are coupled to the pool of oxidizing equivalents available for respiration, rather than anabolic NADPH production.

### 3.3 The sulfo-TAL pathway

The sulfo-TAL (transaldolase) (Liu et al., 2020; Frommeyer et al., 2020) pathway was primarily characterized in Gram-positive Firmicutes, including *Bacillus megaterium* DSM 1804 (Liu et al., 2020) and *Bacillus aryabhattai* (Frommeyer et al., 2020). Subsequent studies have revealed its presence in anaerobic gut microbiota such as *Enterococcus gilvus*, *Clostridium symbiosum*, *Agathobacter rectalis* (formerly known as *Eubacterium rectale*) (Frommeyer et al., 2020) and *Faecalicatena* spp. (Borusak et al., 2024).

**Figure 4:**
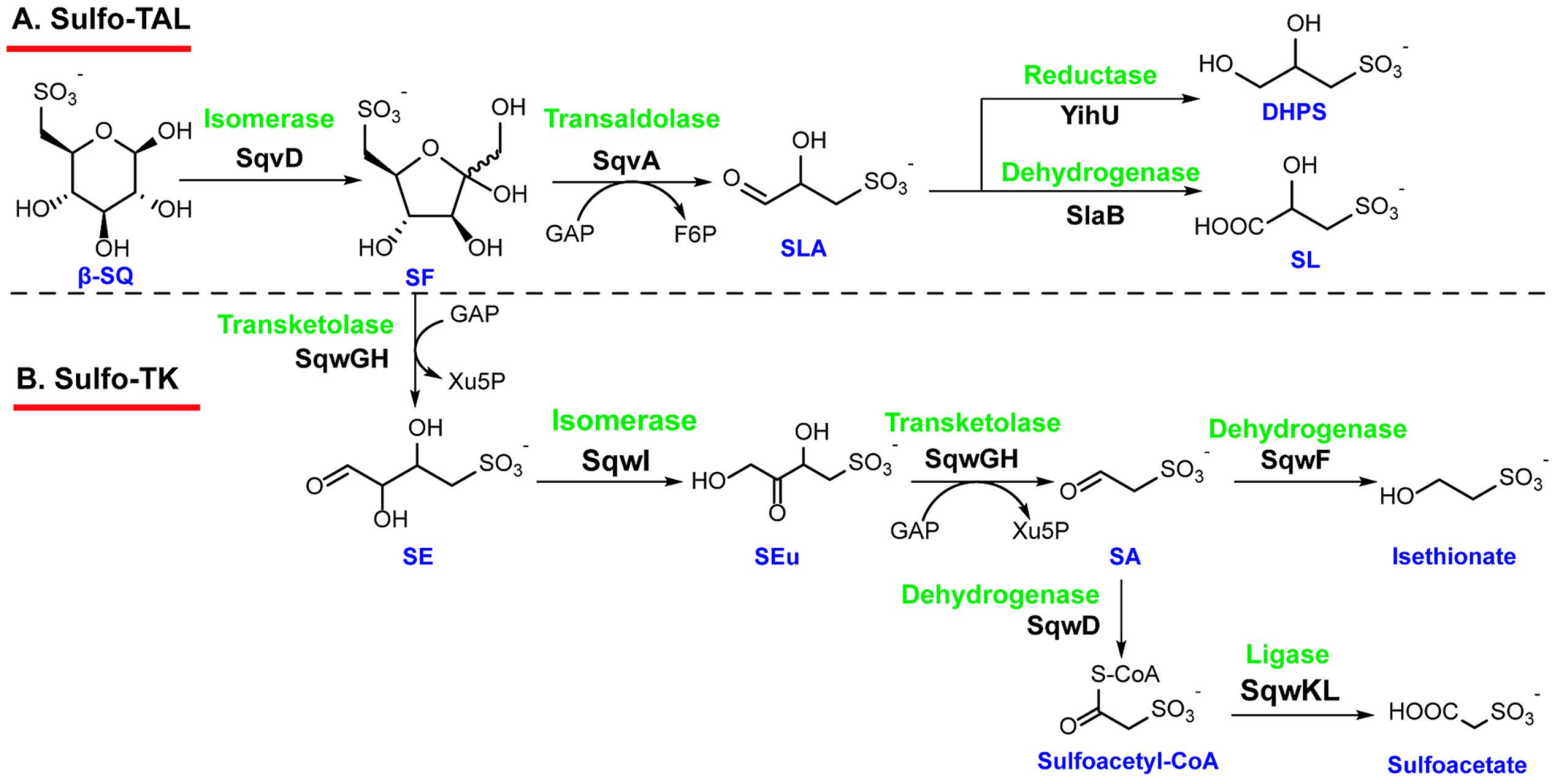
The Sulfo-TAL and Sulfo-TK pathways. **(A)** The sulfo-TAL pathway is designated by its key enayme transaldolase, and it was identified in *Bacillus megaterium* DSM 1804 and *Bacillus aryabhattai*. **(B)** The sulfo-TK pathway is named by its key transketolase, and it was identified in *Clostridium* sp. MSTE9. SE, 4-deoxy-4-sulfoerythrose; SEu, 4-deoxy-4-sulfoerythrulose; SA, sulfoacetaldehyde.

The initial metabolic flux of the sulfo-TAL pathway parallels the sulfo-EMP pathway, wherein SQ is isomerized by SqvD to 6-deoxy-6-sulfo fructose (SF), with a concomitant contribution of the mutarotase SqvB. The subsequent catalytic steps share mechanistic homology with the pentose phosphate pathway. The key transaldolase SqvA, a structural homolog of classical transaldolases, cleaves SF into SLA and a non-sulfonated C3 moiety (dihydroxyacetone). During the reaction, the dihydroxyacetone unit is transferred to glyceraldehyde phosphate (GAP) to yield fructose-6-phosphate (F6P). Cryo-EM analysis of SqvA captures a Schiff base intermediate complex with SF covalently linked to Lys89 (Snow et al., 2023). The resulting SLA is then channeled into distinct metabolic routes: it can be oxidized to SL by sulfolactaldehyde dehydrogenase SlaB in aerobic Bacillus strains, whereas in anaerobic Firmicutes and many Thermotogae and Chloroflexi species, it is reduced to DHPS via sulfolactaldehyde reductase YihU (Figure 4A) (Liu et al., 2020).

### 3.4 The sulfo-TK pathway

Additionally, Liu *et al.* identified the sulfo-TK (transketolase) pathway in *Clostridium* sp. MSTE9. The primary distinction between this pathway and the sulfo-TAL pathway lies in the key lyase enzyme, which is a transketolase instead of a transaldolase. SQ is also initially converted to SF via SqvD, and subsequently, the thiamine pyrophosphate (TPP)-dependent SF transketolase, SqwGH, transfers a C2 ketol unit from SF to the ketol acceptor GAP, yielding 4-deoxy-4-sulfoerythrose (SE) and xylulose-5-phosphate (Xu5P). SE is then isomerized by the isomerase SqwI to form 4-deoxy-4-sulfoerythrulose (SEu). SqwGH subsequently catalyzes a second transketolase reaction, cleaving SEu into sulfoacetaldehyde (SA) via a process analogous to its cleavage of SF. SA is then reduced to isethionate by SqwF, a homolog of metal-dependent alcohol dehydrogenase (ADH) (Figure 4B). Isethionate can be exported from the cell via the sulfite/sulfonate transporter SqwE. It has been revealed that the sulfo-TK pathway is exclusively present in strictly anaerobic bacteria, including various *Clostridia*, *Spirochaetes*, *Thermotogae*, (Liu et al., 2021) and *Faecalicatena* (Borusak et al., 2024).

Notably, some bacteria containing the sulfo-TK pathway lack homologs of SqwF and SqwE. Instead, they have a CoA-acylating sulfoacetaldehyde dehydrogenase SqwD and an ADP-forming sulfoacetate-CoA ligase SqwKL, which oxidized SA to sulfoacetate sequentially, coupled with ATP formation. This sulfo-TK variant distribution among various bacteria, such as *Spirochaetes*, *Firmicutes*, and *Tenericutes* (Chu et al., 2023).

## 4 Metabolism and fate of sulfonated products

For the C-S bond cleavage pathways, SQ is directly desulfurized to yield glucose, which can be further metabolized via glycolysis or other pathways. In contrast, C-C bond cleavage pathways produce short-chain sulfonated carbohydrates, which require additional desulfurization or further degradation steps. When the bacteria capable of C-C bond cleavage pathways were further investigated, it was found that some of them possess not only gene clusters involved in SQ degradation, but also contain genes of the enzymes that can further degrade the short-chain sulfonated carbohydrates. Detailed investigations of these enzymes elucidated the mechanisms, by which these short-chain products are further degraded, desulfurization, and channeled into central metabolism (Cook and Denger, 2002; Cook et al., 2006).

**Figure 5:**
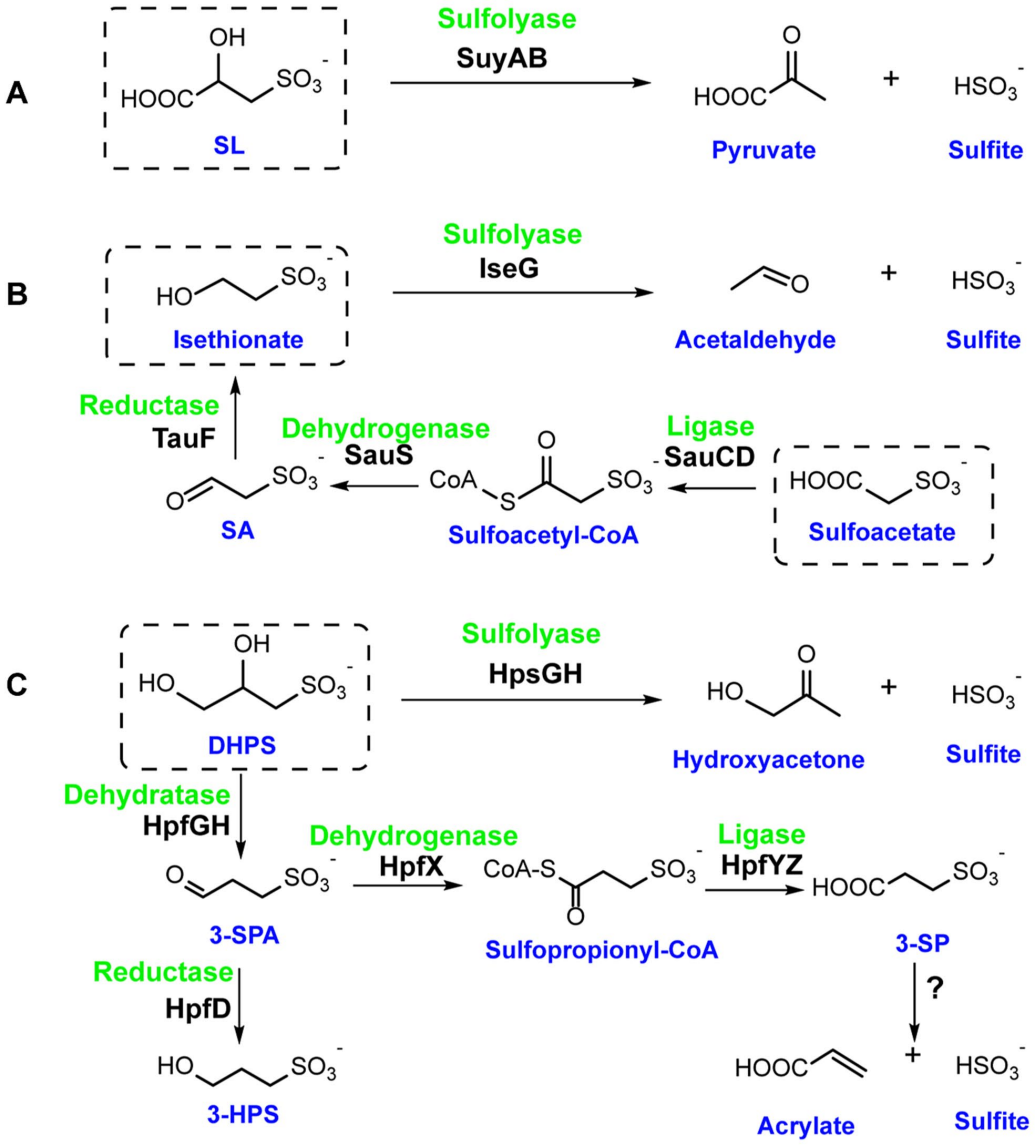
Degradation mechanisms of the sulfonate byproducts sulfolactate (SL), isethionate, sulfoacetate (SA), dihydroxypropanesulfonate (DHPS). **(A)** (*R*)-sulfolactate sulfolyase (SuyAB) was identified in *Cupriavidus pinatubonensis* JMP134. **(B)** Isethionate sulfolyase (IseG), ADP-forming CoA ligase (SauCD), sulfoacetaldehyde dehydrogenase (SauS) and sulfoacetaldehyde reductase TauF were identified in *Bilophila wadsworthia.* **(C)** Dihydroxypropanesulfonate sulfolyase (HpsGH) was identified in *Bilophila wadsworthia.* DHPS dehydratase (HpfGH) was identified in *Klebsiella oxytoca*. 3-sulfopropionaldehyde dehydrogenase (HpfX) and an ADP-forming 3-sulfopropionate-CoA ligase (HpfYZ) were identified in *Enterococcus gilvus*. 3-sulfopropionaldehyde reductase HpfD was identified in *Hungatella hathewayi* and *Enterococcus gilvus*.

It was showed that SL can serve as the sole carbon and energy source for aerobic growth of *Paracoccus pantotrophus* NKNCYSA (Denger et al., 2012; Mikosch et al., 1999; Rein et al., 2005), *Cupriavidus necator* JMP134 (Denger et al., 2012), and *Silicibacter pomeroyi* DSS-3 (Cook et al., 2006; Denger and Smits, 2006). SL could also function as a terminal electron acceptor for the strictly anaerobic bacteria *Bilophila wadsworthia* (Baron, 1997; Laue et al., 1997) and *Desulfitobacterium hafniense* DCB-2 (Cook et al., 2006). SL can be cleaved by the Fe(II)-containing (*R*)-sulfolactate sulfolyase SuyAB (Mayer et al., 2010), yielding sulfite and pyruvate. Isethionate can act as a terminal electron acceptor for anaerobic growth of *Desulfovibrio desulfuricans* IC1, *Desulfitobacterium* spp., *Desulfomicrobium norvegicum*, and *B. wadsworthia* (Cook and Denger, 2002; Lie et al., 1999; Burrichter et al., 2021; Xing et al., 2019). Its degradation is catalyzed by the glycyl radical enzyme (GRE) isethionate sulfolyase (IseG) (Xing et al., 2019), which splits the C-S bond to produce sulfite and acetaldehyde. In *B. wadsworthia*, sulfoacetate can be further converted to isethionate in a way reversing the reactions of the sulfo-TK variant pathway for desulfurization. It is converted to sulfoacetyl-CoA by an ADP-forming CoA ligase SauCD, and then reduced by sulfoacetaldehyde dehydrogenase SauS to form SA. Subsequently, SA is reduced by a reductase TauF to form isethionate, which is cleaved by IseG to produce sulfite and acetaldehyde (Figure 5) (Liu X. et al., 2023).

The DHPS excreted by SQ degradation bacteria can also serve as a terminal electron acceptor for the gut bacterium *B. wadsworthia,* the C-S bond cleavage is mediated by a GRE named dihydroxypropanesulfonate sulfolyase (HpsG), yielding sulfite and hydroxyacetone (Yan and Liu, 2022; Liu et al., 2021; Liu et al., 2020). Interestingly, in some anaerobic gut bacteria, such as *Klebsiella oxytoca* (von Styp Rekowski et al., 2005) and *Enterococcus gilvus*, that contain Sulfo-TAL pathway, DHPS can be further metabolized into 3-sulfopropionaldehyde (3-SPA) by another GRE, the DHPS dehydratase (HpfG) (Liu et al., 2020). 3-SPA is then reduced to 3-hydroxypropanesulfonate (3-HPS) by the 3-sulfopropionaldehyde reductase HpfD and excreted as a metabolic end product (Figure 5). Additionally, in *E. gilvus,* 3-SPA can be oxidized to 3-sulfopropionate (3-SP) by CoA-acylating 3-sulfopropionaldehyde dehydrogenase (HpfX) and an ADP-forming 3-sulfopropionate-CoA ligase (HpfYZ), coupled to the phosphorylation of ADP to ATP (Chen et al., 2024). This process has also been viewed as a Sulfo-TAL extended pathway (Chen et al., 2024). It is noteworthy that 3-SP can also serve as a terminal electron acceptor for anaerobic growth of *B. wadsworthia*. Although the mechanism, by which *B. wadsworthia* degrades 3-SP remains unclear, this example highlights the potential ecological significance of dietary SQ metabolism in shaping the gut microbial community (Chen et al., 2024).

### 4.1 Radical enzymology in anaerobic sulfonate degradation

While aerobic pathways utilize oxygenases (SquD/SqoD) to cleave C-S bonds, strict anaerobes must employ radical chemistry to overcome the high activation energy of sulfonate cleavage in the absence of molecular oxygen. A prominent class of enzymes facilitating this are the Glycyl Radical Enzymes (GREs), which generate a stable protein-based radical to abstract a hydrogen atom from the substrate, enabling difficult rearrangement reactions.

For isethionate desulfonation, the GRE IseG catalyzes the cleavage of the C-S bond in isethionate to yield acetaldehyde and sulfite. This reaction is critical for *B. wadsworthia*, allowing it to use isethionate as a terminal electron acceptor. The mechanism involves the abstraction of a hydrogen atom from C-1 of isethionate, leading to a radical intermediate that fragments to release sulfite. For DHPS cleavage, similarly, the GRE HpsG cleaves DHPS into hydroxyacetone and sulfite (Liu et al., 2020) and represents a major route for H₂S production in the colon (Ijssennagger et al., 2015; Wallace et al., 2018). For DHPS dehydration, in contrast to direct desulfonation, the GRE HpfG found in *Enterococcus gilvus* and *Klebsiella oxytoca* catalyzes the dehydration of DHPS to 3-sulfopropionaldehyde 3-SPA. This radical-mediated dehydration routes the carbon skeleton toward 3-HPS or 3-SP rather than immediate sulfide release, thereby diversifying the metabolic fate of sulfonates in the anoxic gut environment (see Table 1).

**Table 1:** Comparative overview of microbial SQ catabolic pathways: distribution, oxygen requirement, key enzymes, end product and ecological role.

Pathway	Distribution	Oxygen requirement	Key enzymes	End products	Ecological role
C–S Bond cleavage pathways					
Sulfo-ASMO	α/β-Proteobacteria (Gram-negative)	Aerobic	SquD/SmoC; SsuE/SmoA; SquF/SmoB	None (Directly yields glucose)	Complete mineralization
Sulfo-ASDO	γ-Proteobacteria (Gram-negative)	Aerobic	SqoD; SquF	None (Directly yields glucose)	Complete mineralization
C–C Bond cleavage (Sulfoglycolysis)					
Sulfo-EMP	γ-Proteobacteria (Gram-negative) and Actinobacteria (Gram-positive)	Aerobic or Anaerobic	YihS; YihV; YihT; YihU; SlaB	DHPS or SL	DHPS is served as a terminal electron acceptor by some anaerobic bacteria. SL can serve as a carbon source for certain aerobic bacteria and as a terminal electron acceptor for some anaerobic bacteria.
Sulfo-EMP2	Firmicutes (Gram-positive)	Aerobic or Anaerobic	SqvD; SqiK, SqiA; YihU; SlaB	DHPS or SL	
Sulfo-TAL	Firmicutes (Gram-positive), Thermotogae and Chloroflexi (Gram-negative)	Aerobic or Anaerobic	SqvD; SqvA; SlaB; YihU	DHPS or SL	
Sulfo-TK	Firmicutes (Gram-positive), Spirochaetes and Thermotogae (Gram-negative)	Anaerobic	SqvD; SqwGH; SqwI; SqwGH; SqwF; SqwD; SqwKL	Isethionate or sulfoacetate	C2-sulfonates are served as terminal electron acceptors for other anaerobes (e.g., *B. wadsworthia*).
Sulfo-ED	α/β/γ-Proteobacteria (Gram-negative)	Aerobic	SQ-DH; SGL-L; SG-DH; KDSG-A; SlaB	Pyruvate and SL	Pyruvate enters the TCA cycle; SL can be mineralized by other bacteria.

## 5 Dietary sources and environmental loads

Sulfoquinovose (SQ) is estimated to be produced at a rate of 10 billion tons (10 petagrams) annually on a global scale, primarily sequestered within the thylakoid membranes of phototrophs. In the human diet, SQ is ubiquitous in dark green leafy vegetables such as spinach, lettuce (*Lactuca sativa*), and Brassica species (kale, broccoli), where it exists as the headgroup of SQDG. SQDG accounts for approximately 80%–90% of the glycolipid fraction in thylakoid membranes, making it a persistent and high-abundance substrate for the gut microbiome. Edible algae (e.g., nori, kelp) and cyanobacteria are exceptionally rich sources of SQ. In marine environments, these sulfolipids constitute a major fraction of the organic sulfur flux. The constant dietary influx of SQ provides a selective niche for specialized sulfoglycolytic bacteria. Recent studies indicate that dietary SQ availability directly modulates the abundance of *Agathobacter rectalis* (formerly *Eubacterium rectale*) and *Faecalibacterium prausnitzii* in the gut, highlighting its potential as a “sulfobiotic” or prebiotic agent to shape microbiome composition.

## 6 SQ degradation by the microbiota in the gut

SQ is a sulfonated monosaccharide abundant in green leafy vegetables and serves as a selective nutrient for a narrow subset of human gut bacteria. It is primarily fermented by key Firmicutes members, most notably *A. rectalis*, via the sulfo-TAL pathway (Hanson et al., 2021). This process yields acetate and the organosulfonate intermediate DHPS. In a critical interspecies cross-feeding interaction, DHPS is subsequently utilized by sulfite-respiring pathobionts like *B. wadsworthia*. *Bilophila wadsworthia* employs a specialized, oxygen-sensitive glycyl radical enzyme HpsGH to cleave DHPS, generating sulfite which is then reduced to H₂S (Yan and Liu, 2022; Liu et al., 2020). *Agathobacter rectalis* and *Bilophila wadsworthia* cooperate within the human gut microbiota. They utilize recently elucidated metabolic pathways to achieve the interspecies transfer of DHPS, thereby jointly converting plant-derived SQ into H₂S. Metatranscriptomic analyses reveal that the sulfo-TAL pathway is actively and widely expressed across diverse human populations, establishing SQ degradation as a core microbial function in the human gut (Hanson et al., 2021).

The implications of SQ-derived H₂S on gut health are governed by a complex balance between physiological signaling and toxicity, largely dictated by concentration gradients and the local microenvironment. While H₂S functions as a mitochondrial electron donor and cytoprotectant at low physiological concentrations (typically in the micromolar range), elevated levels (millimolar range) can inhibit cytochrome c oxidase and disrupt the colonic mucus barrier (Banerjee, 2011). However, recent multi-omics findings challenge the view that *B. wadsworthia*-derived H₂S is the sole driver of inflammation; rather, inflammation may arise from synergistic metabolic interactions within the microbiome, such as the co-production of ethanol, rather than sulfide accumulation alone (Sayavedra et al., 2025). Recent study suggests that polysulfide species, rather than H₂S itself, play a more critical role in host physiological regulation, as evidenced by research demonstrating the relationship between gut microbiota-derived polysulfides and host function (Uchiyama et al., 2022). Furthermore, host defense mechanisms, including epithelial oxygenation gradients that promote sulfide oxidation and mucin desulfation dynamics, actively modulate this toxicity. Consequently, therapeutic strategies are shifting away from broad microbial suppression toward diet-microbiota-host axis interventions, such as dietary SQ titration or the use of specific prebiotics, to maintain sulfidogenic activity within a safe, physiological window.

Importantly, SQ is not metabolized by the mammalian host, as evidenced by its stability in human cell cultures and the absence of ^13^CO₂ exhalation in germ-free mice gavaged with ^13^C-SQ, highlighting its exclusive role as a microbial substrate. However, fundamental differences exist between human and conventional laboratory mouse gut microbiota in processing SQ. While human gut communities catalyze the complete degradation sequence from SQ to H₂S, the mouse gut microbiome typically performs only the first step. In mice, SQ is predominantly degraded to DHPS by primary degraders such as *E. coli* and *Enterocloster clostridioformis* (Krasenbrink et al., 2025), but further degradation of DHPS to H₂S is markedly absent or minimal. This metabolic discrepancy underscores that the gut microbiota of conventional laboratory mice does not fully recapitulate the human SQ degradation pathway, limiting their utility as models for studying the health impacts of complete SQ metabolism. These insights emphasize the need for caution in translating findings from mouse models and point to the potential of SQ as a prebiotic compound capable of selectively modulating specific, beneficial bacterial populations in the human gut (Krasenbrink et al., 2025).

## 7 Perspective or discussion

As aforementioned, SQ is one of the most abundant organosulfur compounds in the biosphere. Microorganisms that can degrade SQ must be widely spread in different environments. Due to the importance of diet SQ (mainly from green vegetables) and their potential influence on human health, although there have been some investigations on SQ-degradation bacteria from soil and marine (Chen et al., 2021; Liu L. et al., 2023), to date, most of the SQ metabolic studies have been carried out in gut microbes. Upon entering the intestinal tract, SQ cannot be directly used by the host cells, yet it serves as a specialized nutrient source for some gut microbiota (Krasenbrink et al., 2025). The degradation pathway of SQ exhibits a highly specific distribution among human gut microorganisms. Enzymes associated with SQ degradation are encoded in the genomes of nearly half of *A. rectalis*, while being sparsely distributed in other gut bacterial species (Pasolli et al., 2019). In the fecal microbiome, Hanson et al. (2021) revealed that the sulfo-TAL pathway is the predominant route among all SQ degradation pathway, with a relative abundance two orders of magnitude greater than that of the proteobacterial sulfo-EMP pathway. The drivers of these species-specific sulfo-TAL expression patterns are yet to be determined but could involve both dynamic SQ levels in the gut and differences in the enzyme kinetics of SQ-degrading microbes.

Among the aforementioned SQ catabolic pathway, sulfo-TAL pathway is the most frequently observed for microbial SQ degradation in the gut (Hanson et al., 2021). Its key intermediate, DHPS, is further metabolized by various anaerobic pathways among gut microorganisms (Hanson et al., 2021). Specifically, DHPS can be utilized by sulfate and sulfite-reducing bacteria such as *Desulfovibrio* and *B. wadsworthia* as a terminal electron acceptor in respiration, leading to the production of H₂S (Hanson et al., 2021; Burrichter et al., 2018; Xing et al., 2019; Liu et al., 2020; Peck et al., 2019; Barton et al., 2017). Certain fermentative bacteria, including *Klebsiella oxytoca* (von Styp Rekowski et al., 2005) and *Hungatella hathewayi*, are able to reduce DHPS to 3-HPS (Liu et al., 2020). In *E. gilvus*, DHPS is processed via a branched pathway and generates both 3-HPS and 3-SP. And, the latter one could be further utilized by *B. wadsworthia* to produce H₂S (Chen et al., 2024). Both the SQ degradation pathway (primarily attributed to *A. rectalis* and *Faecalibacterium prausnitzii*) and the downstream hydrogen sulfide production pathway (mainly driven by *B. wadsworthia*) are expressed at considerable levels at normal states, underscoring their functional importance.

SQ metabolism exerts dual effects on host health, primarily determined by the balance of its metabolic end-products. On one hand, SQ can be preferentially utilized by beneficial bacteria such as *A. rectalis*, promoting their growth and stimulating the production of short-chain fatty acids (e.g., acetate and butyrate). Krasenbrink et al reported that dietary SQ supplementation can partially rescue the decline in *A. rectalis* populations, highlighting its potential as a candidate prebiotic (Krasenbrink et al., 2025). On the other hand, SQ metabolism may stimulate the overgrowth of bacteria (e.g., *B. wadsworthia*) that may produce excessive H₂S. At a low concentration, H₂S may serve as an antioxidant or a gaseous signaling molecule, contributing positively to host physiology (Olson and Straub, 2016). However, at a high concentration, it may disrupt the intestinal mucus barrier (Ijssennagger et al., 2015), inhibit cellular respiration (Stacy et al., 2021), and potentially promote gut inflammation (Ijssennagger et al., 2016). In addition, hydrogen sulfide production via sulfate reduction may be quantitatively more dominant than SQ-derived pathways in the intestinal environment. Therefore, further investigation into the role of SQ in maintaining gut ecological balance and promoting host health, taking comprehensive factors into account, is warranted.

In summary, significant progresses have been made in understanding microbial SQ degradation in recent years. However, considerable knowledge gaps remain. Given the ubiquitous distribution of SQ in nature, it is anticipated that an increasing number of SQ-degrading bacteria will be identified and characterized in future studies, which will substantially advance our comprehension of the microbial contributions to the global sulfur cycle.
